# Analysis of the interactome of the Ser/Thr Protein Phosphatase type 1 in Plasmodium falciparum

**DOI:** 10.1186/s12864-016-2571-z

**Published:** 2016-03-17

**Authors:** Thomas Hollin, Caroline De Witte, Astrid Lenne, Christine Pierrot, Jamal Khalife

**Affiliations:** Center for Infection and Immunity of Lille, Inserm U1019-CNRS UMR 8204, University of Lille Nord de France, Institut Pasteur de Lille, 1 Rue du Professeur Calmette, Lille, France

**Keywords:** *Plasmodium*, PP1, Interactome, RVxF motif

## Abstract

**Background:**

Protein Phosphatase 1 (PP1) is an enzyme essential to cell viability in the malaria parasite *Plasmodium falciparum* (Pf). The activity of PP1 is regulated by the binding of regulatory subunits, of which there are up to 200 in humans, but only 3 have been so far reported for the parasite. To better understand the *P. falciparum* PP1 (PfPP1) regulatory network, we here report the use of three strategies to characterize the PfPP1 interactome: co-affinity purified proteins identified by mass spectrometry, yeast two-hybrid (Y2H) screening and *in silico* analysis of the *P. falciparum* predicted proteome.

**Results:**

Co-affinity purification followed by MS analysis identified 6 PfPP1 interacting proteins (Pips) of which 3 contained the RVxF consensus binding, 2 with a Fxx[RK]x[RK] motif, also shown to be a PP1 binding motif and one with both binding motifs. The Y2H screens identified 134 proteins of which 30 present the RVxF binding motif and 20 have the Fxx[RK]x[RK] binding motif. The *in silico* screen of the Pf predicted proteome using a consensus RVxF motif as template revealed the presence of 55 potential Pips. As further demonstration, 35 candidate proteins were validated as PfPP1 interacting proteins in an ELISA-based assay.

**Conclusions:**

To the best of our knowledge, this is the first study on PfPP1 interactome. The data reports several conserved PP1 interacting proteins as well as a high number of specific interactors to PfPP1. Their analysis indicates a high diversity of biological functions for PP1 in *Plasmodium*. Based on the present data and on an earlier study of the Pf interactome, a potential implication of Pips in protein folding/proteolysis, transcription and pathogenicity networks is proposed. The present work provides a starting point for further studies on the structural basis of these interactions and their functions in *P. falciparum*.

**Electronic supplementary material:**

The online version of this article (doi:10.1186/s12864-016-2571-z) contains supplementary material, which is available to authorized users.

## Background

Malaria continues to be a major health problem and a leading cause of child mortality of the inter-tropical regions despite extensive research efforts. The present situation is also increasingly complicated by the emergence of parasite resistance to multiple drugs much more rapidly than the development of novel anti-malarials. A major obstacle in devising new control tools is our limited knowledge of basic parasite biology and the paucity of identified potential intervention targets. *Plasmodium* species are obligate intracellular protozoan parasites that undergo a number of developmental stages in the vertebrate host and the invertebrate vector. During the last decade, several studies strongly indicated that protein phosphorylation and dephosphorylation processes, governed by kinases and phosphatases respectively, play a central and essential role in *Plasmodium* cell cycle and developmental regulation [1, 2].

In *Plasmodium falciparum* (Pf), the most deadly apicomplexan parasite, efforts have begun for an examination of the biological roles of protein kinases and phosphatases and their potential as drug targets. In this context, biochemical and cloning studies have reported enzymatic activities and the genes of catalytic subunits related to plasmodial phosphatases. These include PfPP1c, PfPP2A, PfPP2B and PfPP2C, which have been shown to be highly conserved during evolution, suggesting an essential role for these enzymes [3–5]. With respect to the Pf Protein Phosphatase type 1 catalytic subunit or PfPP1c, it cannot be considered *per se* as a suitable drug target (> 80 % identity with human and yeast PP1 or GLC7). Indeed, complementation studies showed that PfPP1 was able to rescue yeast mutated in PP1 [6]. In *P. falciparum* functional studies suggested that its activity predominates over the other phosphatases in blood parasites [7]. Finally, phenotypic PfPP1 gene knockdown and the use of phosphatase inhibitors indicated that it seems to be essential for parasite asexual development in erythrocytes [2, 4]. At this stage, it is important to note that free PP1c seems to be toxic for the cell [8] but a considerable number of proteins interacting with PP1c have been reported that direct the localization, specificity and the level of its activity in yeast and higher eukaryotic cells [9]. Indeed, about 200 proteins have been shown to physiologically interact with PP1c, constituting a ‘PP1c platform’ that provides a framework offering diverse functions to this enzyme in cell division, transcription and apoptosis for instance [10]. Structural and functional studies revealed that the interaction of regulators with PP1 involved several binding sites, including mainly the so-called RVxF and SILK motifs [8, 11]. The most studied binding motif is the RVxF for which it has been established a first short consensus sequence as [RK]x_0-1_[VI]{P}[FW] where x could be any amino acid and {P} any amino acid except P [12].

The importance of these proteins in the regulation of PP1 activity in vitro and in vivo prompted us to identify the regulators of PfPP1c and to address their functions. Our initial focus based on the completion of the Pf genome and the delineation of known PP1 regulators has revealed the existence of four conserved regulators. So far, three gene products, Pf Leucine Rich Repeat 1 (PfLRR1), Pf Inhibitor 2 (PfI2) and Pf Inhibitor 3 (PfI3) have been thoroughly explored to define their functions in this parasite. Biochemical and structure-activity relationship studies demonstrated that these regulators indeed bind to PfPP1. In these studies, we showed that *P. falciparum* PP1 is submitted to a control of its activity by PfLRR1 (a homolog of yeast SDS22), Inhibitor-2 (PfI2) and -3 (PfI3) with substantial differences compared to the human orthologs for Inhibitor 2 and 3 [13–18]. PfI2 exhibits an inhibitory role on PfPP1 activity, the short RVxF binding motif, not present in human I2, a secondary Fxx[RK]x[RK] binding motif and a peptide sequence 30 % shorter than its human homolog. PfI3, although it contains the RVxF consensus motif, does not seem to be an inhibitor but rather an activator of PfPP1 in vitro and is unable to complement I3 deficient yeast, suggesting a specific role for PfI3 on PfPP1. In *P. falciparum*, our reverse genetic analyses suggest that PfI2 as well as PfI3 are essential for parasite growth. Further, NMR analysis showed that the main domain of PfI3 binding to PfPP1 is within residues P^39^-W^45^. The mutation of the RVxF motifs in both proteins significantly affects the binding to PP1. Finally, peptides derived from these binding motifs have been shown to inhibit *P. falciparum* growth in vitro [17, 18].

These observations underscore the importance of PP1 regulators and promote an increased interest to identify *P. falciparum* PP1 interacting proteins (Pips). In the present work, biochemical approaches, yeast two-hybrid screens and *in silico* analysis were used to define the Pips that are the starting point to elucidate PP1 interaction networks in *P. falciparum* and to provide new pathways for further studies to reveal potential targets for specific pharmacological intervention.

## Results and discussion

### Identification of Pips by affinity/Mass Spectrometry

In order to identify Pips expressed by *P. falciparum*, affinity purification on PfPP1 followed by mass spectrometry was carried out. In this approach, soluble extracts from blood stage parasites cleared as described in Materials and Methods were incubated with PfPP1 covalently bound to sepharose beads. The eluate was separated by SDS-PAGE and the proteins detected by coomassie blue staining were identified by MS-MS. As shown in Table 1, only 6 *P. falciparum* proteins were clearly identified based on the number of peptides analysed and the corresponding mascot scores for each protein (Additional file 1: Table S1). The identified proteins, Ornithine aminotransferase, GAPDH and Phosphoethanolamine N-methyltransferase contained the motifs KTVKF, KLVSW and RIIF respectively, which comply with the RVxF consensus sequence, while PfHSP90 and Pf disulfide isomerase have only one putative Fxx[RK]x[RK] motif (FENRKK and FNKKNK respectively). Sequence analyses of PfHSP70 showed 2 potential binding motifs RLVNF and FKRKNR which correspond to RVxF and Fxx[RK]x[RK] motifs respectively, supporting a direct interaction. However, it could not be excluded that this interaction may be related to the chaperone functions of HSPs. Hence, it would be necessary to further dissect the role of HSPs on the function of PP1. Previous studies reported that PP1 interacts with murine and human HSP70 [19, 20] which are in agreement with the above data. Further, amino acid sequence analysis of PfHSP70 showed 70 % identity with mouse and human HSP70 and 2 potential binding motifs, however only the binding motif Fxx[RK]x[RK] is present in the latter (Additional file 2: Table S2). Overall, the above approach led to the detection of a low number of direct or indirect Pips when compared to the high number of reported regulators/substrates of PP1c in other species. The low recovery by the affinity/mass spectrometry analysis could be attributed to several factors including the presence of very few free Pips in parasite extracts, their low solubility in buffers suitable for affinity column and/or the competition between Pips.

**Table 1:** Pips identified by affinity chromatography/mass spectrometry

Band^a^	PlasmoDB	Name^c^	Motifs (Position a.a.)^d^
	Accession number^b^		
1	PF3D7_0708400	heat shock protein 90 (HSP90)	FENRKK (371–376)
2	PF3D7_0818900	heat shock protein 70 (HSP70)	RLVNF (248–252)
			FKRKNR (257–262)
3	PF3D7_0827900	protein disulfide isomerase (PDI8)	FNKKNK (112–117)
4	PF3D7_0608800	ornithine aminotransferase (OAT)	KTVKF (405–409)
5	PF3D7_1462800	glyceraldehyde-3-phosphate dehydrogenase (GAPDH)	KLVSW (312–316)
6	PF3D7_1343000	phosphoethanolamine N-methyltransferase (PMT)	KIIF (103–106)

^a^Number of stained bands on SDS-PAGE

^b^Annotations available on PlasmoDB database (v24)

^c^The identified proteins were detected in 2 independent proteins extracts

^d^Sequences complying with consensus of RVxF and Fxx[RK]x[RK] were indicated

### Yeast two hybrid screening of a Pf cDNA library

To further detect additional Pips in *P. falciparum*, a cDNA library was screened in a yeast two hybrid (Y2H) system using full-length PfPP1c as bait and clones growing on high stringency selection media to reduce the number of false positive clones. Five independent screens yielded 189 clones from a total of 3.3 × 10^6^ clones screened. After sequencing, all clones showed open reading frames encoding Pf genes with 53 clones in frame with GAL4AD and 136 clones out of frame. Amino acid sequence comparison revealed that 8 proteins in frame with GAL4AD were also found among the out-of-frame clones. These data are in agreement with an earlier observation indicating that out-of-frame proteins could be considered as valid interactors in Y2H screening [21] and with recent results suggesting that translational frameshift could be means for yeast to reduce the level of expression of exogenous proteins in order to grow on selective media. Indeed, this was observed with DHFR where most of isolated clones were not in frame with GAL4AD. In addition, this work showed that a reduction of growth rate of yeast was observed when the screening was carried out with DHFR fused in frame with GAL4AD [22]. Consequently, the GAL4AD in frame and out-of-frame clones were included in our analysis. In total, sequences identified from 189 clones represented 134 different proteins (Table 2) of which 27 were obtained more than once with 10 clones for PF3D7_1202600 and 9 clones for PF3D7_0919900 (Additional file 3: Table S3). Analysis of the sequences revealed that HSP70 (PF3D7_0818900) was isolated by Y2H screens, reinforcing the idea of its interaction with PfPP1 that we observed using parasite extracts and a PfPP1 affinity column. This supports the interaction of HSP70 with PP1 and suggests that besides its chaperone function it may control PP1 activity and/or regulate its binding with other partners. The Y2H screens also identified the gene PF3D7_1020900 as a Pip. Its gene product shares 72 % identity with a yeast ADP Ribosylation Factor (ARF1) which has been shown to interact with yeast PP1 [23] (Additional file 2: Table S2). Analysis of the Pf isolated fragment did not show the presence of known binding motifs to PP1. However the analysis of the full length sequence revealed the presence of 1 RVxF motif, suggesting the presence of at least 2 PfPP1 potential binding motifs in this protein. Further, PF3D7_1103100 and PF3D7_1460700 showed 31 and 39 % of identity with yeast RPP1B and RPL27A respectively (Additional file 2: Table S2), identified by different approaches as interactors of yeast PP1 [24, 25].

**Table 2:** Pips identified by Yeast two-hybrid screening

	PlasmoDB Accession number^a^	Name^a^	Growth on selective plates (number of isolated clones)^b^	RVxF motifs^c^
In frame genes	PF3D7_0107600	serine/threonine protein kinase, putative	TDO/A (1)	
	PF3D7_0220000	liver stage antigen 3 (LSA3)	QDO/A (2)	KKVRF
	PF3D7_0418300	conserved Plasmodium protein, unknown function	QDO/A (1)	
	PF3D7_0520800	conserved Plasmodium protein, unknown function	TDO/A (1)	
	PF3D7_0520900	S-adenosyl-L-homocysteine hydrolase (SAHH)	TDO/A (1)	
	PF3D7_0610100	Pre-mRNA-splicing factor SLU7, putative (SLU7)	TDO/A (1)	
	PF3D7_0611800	conserved Plasmodium protein, unknown function	TDO/A (1)	
	PF3D7_0613800	transcription factor with AP2 domain(s) (ApiAP2)	QDO/A (1)	RGVYF
			TDO/A (1)	
	PF3D7_0623100	nuclear polyadenylated RNA-binding protein NAB2, putative	TDO/A (2)	
	PF3D7_0718100	exported serine/threonine protein kinase (EST)	TDO/A (1)	
	PF3D7_0720700	phosphoinositide-binding protein, putative	TDO/A (1)	KIKF
	PF3D7_0724600	protein kinase, putative	TDO/A (1)	
	PF3D7_0803400	DNA repair protein rad54, putative	QDO/A (1)	
	PF3D7_0816600	ClpB protein, putative (ClpB1)	TDO/A (1)	
	PF3D7_0917900	heat shock protein 70 (HSP70-2)	TDO/A (1)	
	PF3D7_0919900	regulator of chromosome condensation, putative	QDO/A (8)	KSVSF
	PF3D7_1008000	histone deacetylase 2 (HDA2)	TDO/A (1)	
	PF3D7_1008100	conserved Plasmodium protein, unknown function	TDO/A (1)	KSVSF
	PF3D7_1020900	ADP-ribosylation factor (ARF1)	TDO/A (1)	
	PF3D7_1031600	conserved Plasmodium protein, unknown function (GEXP15)	QDO/A (3)	KKVQF
	PF3D7_1105100	histone H2B (H2B)	TDO/A (1)	
	PF3D7_1130700	structural maintenance of chromosome protein, putative	TDO/A (2)	
	PF3D7_1202600	conserved protein, unknown function	QDO/A (10)	KNVTF/KCVSF/KQVTF/RTVSF/KKVTF/KSVSF/KKVTF
	PF3D7_1205500	zinc finger protein, putative	TDO/A (1)	
	PF3D7_1228600	merozoite surface protein 9 (MSP9)	TDO/A (1)	
	PF3D7_1229600	conserved Plasmodium protein, unknown function	TDO/A (1)	
	PF3D7_1303800	conserved Plasmodium protein, unknown function	TDO/A (1)	
	PF3D7_1317600	conserved Plasmodium protein, unknown function	TDO/A (1)	KYIYF
	PF3D7_1325800	conserved Plasmodium protein, unknown function	TDO/A (1)	
	PF3D7_1346100	protein transport protein SEC61 subunit alpha (SEC61)	TDO/A (1)	KGIEF
	PF3D7_1358200	conserved Plasmodium protein, unknown function	TDO/A (1)	
Out of frame genes	mal_mito_2	cytochrome c oxidase subunit 1 (COX1)	TDO/A (2)	
	PF3D7_0110200	FAD-linked sulfhydryl oxidase ERV1, putative (ERV1)	TDO/A (1)	KINF
	PF3D7_0202400	conserved Plasmodium protein, unknown function	TDO/A (1)	
	PF3D7_0205700.1	conserved Plasmodium protein, unknown function	TDO/A (1)	
	PF3D7_0207100	conserved Plasmodium protein, unknown function	TDO/A (1)	
	PF3D7_0207500	serine repeat antigen 6 (SERA6)	TDO/A (1)	
	PF3D7_0307700	conserved Plasmodium protein, unknown function	TDO/A (1)	
	PF3D7_0310400	parasite-infected erythrocyte surface protein (PIESP1)	TDO/A (1)	
	PF3D7_0312800	60S ribosomal protein L26, putative	TDO/A (1)	
	PF3D7_0317300	conserved Plasmodium protein, unknown function	TDO/A (1)	
	PF3D7_0319500	RNA binding protein, putative	TDO/A (1)	
	PF3D7_0407700	conserved Plasmodium protein, unknown function	TDO/A (1)	RDIIF
	PF3D7_0417400	conserved Plasmodium protein, unknown function	TDO/A (1)	
	PF3D7_0418000	conserved Plasmodium protein, unknown function	TDO/A (2)	
	PF3D7_0418300	conserved Plasmodium protein, unknown function	TDO/A (2)	
	PF3D7_0419900	phosphatidylinositol 4-kinase, putative	QDO/A (2)	
			TDO/A (1)	
	PF3D7_0420000	zinc finger protein, putative	TDO/A (1)	
	PF3D7_0509200	leucine-rich repeat protein (LRR2)	QDO/A (1)	
	PF3D7_0516600	translation initiation factor IF-2	TDO/A (1)	
	PF3D7_0516900	60S ribosomal protein L2 (RPL2)	TDO/A (1)	KVIF
	PF3D7_0520800	conserved Plasmodium protein, unknown function	QDO/A (1)	
	PF3D7_0520900	S-adenosyl-L-homocysteine hydrolase (SAHH)	TDO/A (1)	
	PF3D7_0525200	conserved Plasmodium protein, unknown function	TDO/A (2)	KIEF/KDVLF
	PF3D7_0530000	conserved Plasmodium protein, unknown function	TDO/A (1)	
	PF3D7_0532100	early transcribed membrane protein 5 (ETRAMP5)	TDO/A (2)	
	PF3D7_0602000	conserved Plasmodium protein, unknown function	TDO/A (1)	
	PF3D7_0604500	conserved Plasmodium protein, unknown function	TDO/A (1)	
	PF3D7_0611700	60S ribosomal protein L39 (RPL39)	TDO/A (2)	
	PF3D7_0613800	transcription factor with AP2 domain(s) (ApiAP2)	TDO/A (1)	
	PF3D7_0617200	conserved Plasmodium protein, unknown function	QDO/A (1)	KQIGF
	PF3D7_0617800	histone H2A (H2A)	QDO/A (2)	
			TDO/A (6)	
	PF3D7_0625300	DNA polymerase 1, putative	TDO/A (1)	
	PF3D7_0709300	Cg2 protein (CG2)	TDO/A (1)	KVNF
	PF3D7_0719900	conserved Plasmodium membrane protein, unknown function	TDO/A (1)	
	PF3D7_0721100	conserved Plasmodium protein, unknown function	QDO/A (1)	
			TDO/A (1)	
	PF3D7_0725300	conserved Plasmodium protein, unknown function	TDO/A (1)	
	PF3D7_0806900	conserved Plasmodium protein, unknown function	QDO/A (1)	KNIGF/KIRW
	PF3D7_0809400	conserved Plasmodium protein, unknown function	TDO/A (1)	
	PF3D7_0811400	conserved protein, unknown function	TDO/A (1)	
	PF3D7_0813200	CS domain protein, putative	TDO/A (1)	
	PF3D7_0813300	conserved Plasmodium protein, unknown function	TDO/A (1)	
	PF3D7_0814000	60S ribosomal protein L13-2, putative	QDO/A (2)	RVNF
	PF3D7_0815800	vacuolar sorting protein VPS9, putative	TDO/A (1)	
	PF3D7_0818000	conserved protein, unknown function	TDO/A (1)	
	PF3D7_0818900^d^	heat shock protein 70 (HSP70)	TDO/A (1)	
	PF3D7_0831700	heat shock protein 70, putative (HSP70-x)	QDO/A (1)	
	PF3D7_0904000	GTPase-activating protein, putative	TDO/A (1)	
	PF3D7_0919900	regulator of chromosome condensation, putative	TDO/A (1)	
	PF3D7_0921700	conserved Plasmodium protein, unknown function	QDO/A (1)	
	PF3D7_0924100	conserved Plasmodium protein, unknown function	TDO/A (1)	
	PF3D7_0932100	protein MAM3, putative	TDO/A (1)	
	PF3D7_0933300	conserved Plasmodium protein, unknown function	TDO/A (1)	
	PF3D7_1020200	conserved Plasmodium protein, unknown function	TDO/A (1)	KNVFF
	PF3D7_1023400	HORMA domain protein, putative	QDO/A (1)	
	PF3D7_1029900	conserved Plasmodium protein, unknown function	TDO/A (1)	
	PF3D7_1032000	ribosome maturation factor RimM, putative (RimM)	TDO/A (1)	
	PF3D7_1103100	60S acidic ribosomal protein P1, putative (RPP1)	TDO/A (1)	
	PF3D7_1105100	histone H2B (H2B)	TDO/A (2)	
	PF3D7_1107300	polyadenylate-binding protein-interacting protein 1, putative (PAIP1)	TDO/A (1)	
	PF3D7_1108600	endoplasmic reticulum-resident calcium binding protein (ERC)	TDO/A (1)	KVYF
	PF3D7_1116200.1	pyridoxine biosynthesis protein PDX2 (PDX2)	QDO/A (1)	
	PF3D7_1117700	GTP-binding nuclear protein ran/tc4 (RAN)	QDO/A (1)	RQIQF
	PF3D7_1121600	circumsporozoite-related antigen,exported protein 1 (EXP1)	TDO/A (1)	
	PF3D7_1127900	conserved Plasmodium protein, unknown function	TDO/A (1)	
	PF3D7_1141400	phosphatidylinositol N-acetylglucosaminyltransferase subunit H, putative (PIGH)	TDO/A (1)	
	PF3D7_1149000	antigen 332, DBL-like protein (Pf332)	TDO/A (1)	KIIW
	PF3D7_1206800	conserved Plasmodium protein, unknown function	QDO/A (1)	
	PF3D7_1210600	conserved Plasmodium protein, unknown function	QDO/A (1)	
	PF3D7_1211200	conserved Plasmodium protein, unknown function	TDO/A (1)	KVKF/KRINF/KYIEF
	PF3D7_1214100	GPI ethanolamine phosphate transferase 3, putative (PIGO)	TDO/A (1)	
	PF3D7_1222300	endoplasmin, putative (GRP94)	TDO/A (1)	
	PF3D7_1224000	GTP cyclohydrolase I (GCH1)	TDO/A (1)	
	PF3D7_1227000	conserved Plasmodium protein, unknown function	TDO/A (2)	
	PF3D7_1227700	conserved Plasmodium protein, unknown function	TDO/A (1)	
	PF3D7_1228600	merozoite surface protein 9 (MSP9)	QDO/A (1)	
			TDO/A (1)	
	PF3D7_1229400	macrophage migration inhibitory factor (MIF)	QDO/A (1)	
			TDO/A (1)	
	PF3D7_1231600	pre-mRNA-splicing factor ATP-dependent RNA helicase PRP2, putative (PRP2)	TDO/A (1)	
	PF3D7_1234600	conserved Plasmodium protein, unknown function	TDO/A (1)	
	PF3D7_1234900	conserved Plasmodium protein, unknown function	TDO/A (1)	
	PF3D7_1240600	erythrocyte membrane protein 1, PfEMP1 (VAR)	TDO/A (1)	
	PF3D7_1249800	THO complex subunit 2, putative (THO2)	TDO/A (1)	
	PF3D7_1303800	conserved Plasmodium protein, unknown function	TDO/A (2)	
	PF3D7_1306400	26S protease regulatory subunit 10B, putative (RPT4)	TDO/A (1)	
	PF3D7_1308400	conserved Plasmodium protein, unknown function	QDO/A (1)	RRVLF
	PF3D7_1308800	tyrosine recombinase (INT)	QDO/A (1)	KYIKF
			TDO/A (1)	
	PF3D7_1316100	inositol polyphosphate kinase, putative (IPK2)	TDO/A (1)	
	PF3D7_1324800	dihydrofolate synthase/folylpolyglutamate synthase (DHFS-FPGS)	QDO/A (1)	
	PF3D7_1326600	conserved Plasmodium protein, unknown function	QDO/A (2)	
	PF3D7_1336600	conserved Plasmodium protein, unknown function	TDO/A (1)	
	PF3D7_1342300	tetratricopeptide repeat family protein, putative	TDO/A (1)	
	PF3D7_1342900	transcription factor with AP2 domain(s) (ApiAP2)	TDO/A (1)	
	PF3D7_1346400	conserved Plasmodium protein, unknown function	TDO/A (1)	
	PF3D7_1348400	conserved Plasmodium membrane protein, unknown function	TDO/A (1)	
	PF3D7_1351200	conserved Plasmodium protein, unknown function	TDO/A (1)	KYVNW
	PF3D7_1356900	protein kinase 5 (PK5)	TDO/A (1)	
	PF3D7_1357400	conserved Plasmodium protein, unknown function	QDO/A (1)	KSIRF
	PF3D7_1360700	E3 SUMO-protein ligase PIAS, putative (PIAS)	QDO/A (1)	KKVLW
	PF3D7_1361200	conserved Plasmodium protein, unknown function	TDO/A (1)	
	PF3D7_1366400	rhoptry protein (Rhop148)	TDO/A (1)	KILF
	PF3D7_1417600	conserved Plasmodium protein, unknown function	TDO/A (1)	
	PF3D7_1423700	conserved Plasmodium protein, unknown function	TDO/A (1)	
	PF3D7_1444300	1-acyl-sn-glycerol-3-phosphate acyltransferase, putative (LPAAT)	TDO/A (1)	
	PF3D7_1456500	conserved Plasmodium protein, unknown function	QDO/A (2)	
	PF3D7_1457000	signal peptide peptidase (SPP)	QDO/A (1)	
	PF3D7_1460500	conserved Plasmodium protein, unknown function	TDO/A (1)	KVDF/KTVSF
	PF3D7_1460700	60S ribosomal protein L27 (RPL27)	TDO/A (1)	
	PF3D7_1464500	conserved Plasmodium membrane protein, unknown function	TDO/A (1)	
	PF3D7_1465900	40S ribosomal protein S3, putative	TDO/A (1)	
	PF3D7_1466300	26S proteasome regulatory subunit RPN2, putative (RPN2)	TDO/A (1)	
	PF3D7_1470800	conserved Plasmodium protein, unknown function	TDO/A (1)	
	PF3D7_1471900	conserved Plasmodium protein, unknown function	TDO/A (1)	KIIEF

^a^Annotations available on PlasmoDB database (v24)

^b^Selection medium used for the growth of isolated yeast clones were indicated. Numbers indicate the count of isolated clones. *TDO/A* Triple dropout supplemented with Aureobasidin A, *QDO/A* Quadruple dropout supplemented with Aureobasidin A

^c^The presence and the sequence of RVxF binding motifs were indicated

^d^Shared proteins isolated by affinity purification and Y2H screening

When the identified sequences were analyzed for the presence of known binding motifs to PP1, 30 proteins share the RVxF binding motif and 20 contain the Fxx[RK]x[RK] motif, supporting the idea of their role as potential regulators of PP1. Indeed, it was reported that 70 % of PP1 interacting proteins containing the RVxF motif are regulators of PP1 activity [26].

Further examination of Y2H screening revealed that some identified clones correspond to different portions of the same protein (16 clones representing 7 proteins). For instance, PF3D7_1303800 interacts with PfPP1 through the protein portions 4987–5278, 6176–6407 (containing the Fxx[RK]x[RK] motif) and 9165–9271 while PF3D7_0419900 binds PfPP1 via the portions 1857–1998 and 4414–4679 (Additional file 3: Table S3). For these proteins it seems that their binding to PfPP1 could take place through at least 2 to 3 regions, potentially involving novel binding motifs.

Among the isolated clones, 8 cDNAs representing 6 known kinases were identified as potential interacting proteins for PP1 but none of the fragments contains any known binding motifs. This supports the idea that these kinases encompass new PP1-binding region/motifs. Examination of the full length proteins of these 6 kinases showed the presence of at least one binding motif (Additional file 3: Table S3). It would be important to check whether these motifs are also involved in interaction with PP1. Taken together, it could be speculated that these kinases could be direct substrates and/or regulators of PfPP1 and vice versa.

In addition to the interaction of PfPP1 and kinases, PfH2A, PfH2B and Pf Histone deacetylase 2 (PfHDA2) have been identified as Pips. All of these proteins are associated with nucleosome modifications and could direct the control of gene expression. Previous studies showed that the phosphorylation marks of H2A and H2B are detected along with lysine acetylation [27, 28]. The interaction of H2A and PP1 has also been reported in yeast [24, 29] (Additional file 2: Table S2). Taken together, these observations suggest a potential role and/or a control of the PP1 network in the marks of histones to govern diverse nuclear actions in *P. falciparum*.

### Identification of Pips by *in silico* screening of the Pf genome

**Fig. 1:**
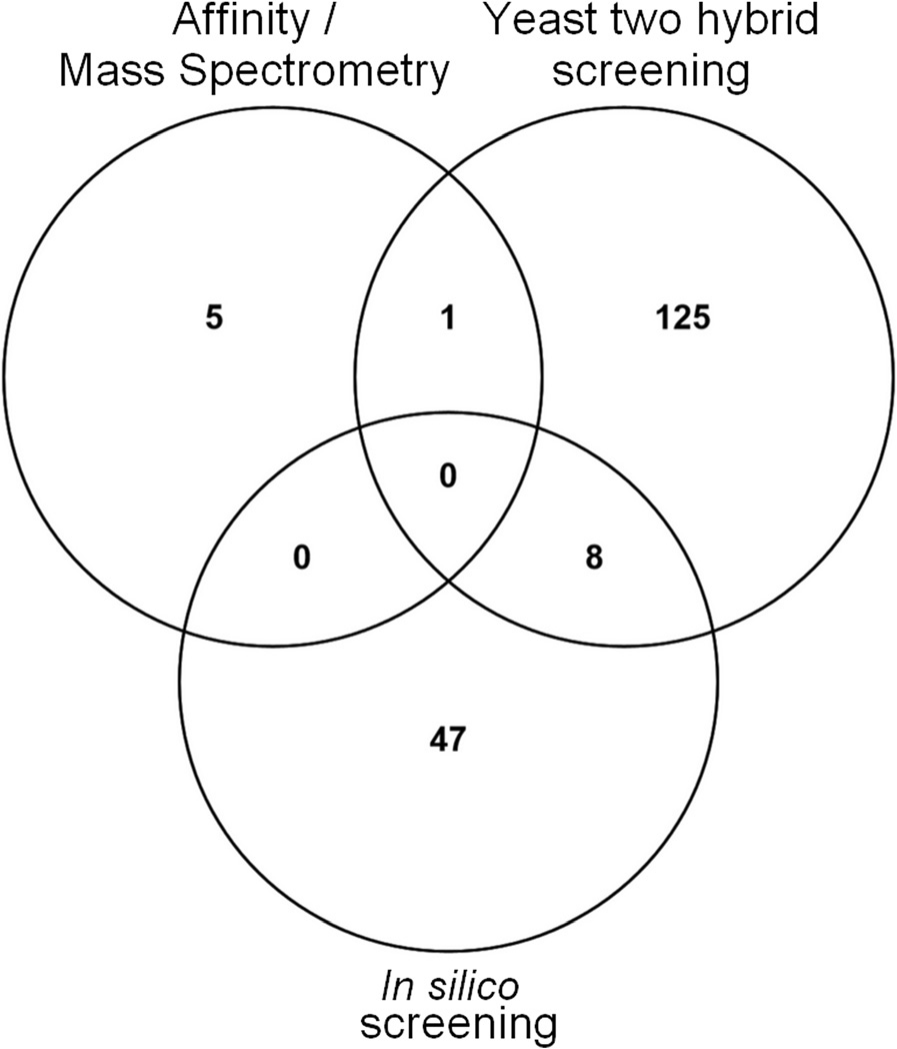
Venn diagram. Graphic shows the distribution of identified Pips by the three approaches

PP1 partners have been identified by biochemical and Y2H approaches, both of which can have their limitations: the quality and quantity of protein preparations, or the incapacity of yeast to produce Pf proteins (or proteins toxic for the growth of yeast). In order to further examine the presence of Pips genes in Pf, we used an extended RVxF consensus sequence mentioned in Materials and Methods. The choice of this extended consensus sequence, referred thereafter as RVxF^ext^, was based on previous studies indicating its high specificity [30, 31]. The first consensus sequence defined by Wakula et al. [12] was not used in this study because it is less restrictive and it occurs randomly in ~25 % of all proteins [30]. The bioinformatics analysis was performed with ORFs present in PlasmoDB (v24). This screening showed the presence of 55 RVxF^ext^ motif containing proteins in *P. falciparum* of which 5 proteins contain at least 2 motifs (Table 3). Two RVxF^ext^ containing proteins were excluded as one corresponds to a pseudogene and the second is annotated twice in PlasmoDB. Interestingly, among the 55 *in silico* Pips, 42 exhibit at least one additional binding motif to PP1 (SILK and/or Fxx[RK]x[RK]) (Additional file 4: Table S4). In addition, 8 proteins identified *in silico* were also isolated by Y2H screening while 1 protein was common between the affinity/MS-MS approach and Y2H screening (Fig. 1). Among the 8 shared proteins, 6 clones identified by Y2H screening followed the RVxF^ext^ consensus sequence. Further, homologs to 2 potential *in silico* PfPips (PF3D7_1107700, PF3D7_1220100) have been shown to interact with PP1 in *S. cerevisiae* (Additional file 2: Table S2) [23, 32, 33]. The *in silico* screening applied in this work cannot include PfLRR1 and Pf Inhibitors 2 and 3, known to bind and to regulate PfPP1 activity. Indeed, PfLRR1 binds PP1 through LRR motifs, however PfI2 and PfI3 encompass the short RVxF motif.

**Table 3:** Pips identified by genome *in silico* analysis

PlasmoDB Accession number^a^	Name^a^	RVxF^ext^ motifs^b^
PF3D7_0204600	conserved Plasmodium protein, unknown function	RKSVRW
PF3D7_0214400	conserved Plasmodium protein, unknown function	KKKVTF
PF3D7_0220000^c^	liver stage antigen 3 (LSA3)	KKKVRF
PF3D7_0304300	conserved Plasmodium protein, unknown function	KKKVTF
PF3D7_0305500	conserved Plasmodium protein, unknown function	KRNVSF/KRSVHF
PF3D7_0323300	conserved protein, unknown function	KKSVSF
PF3D7_0404700	dipeptidyl aminopeptidase 3 (DPAP3)	RKNVTF
PF3D7_0407300	transcription factor, putative	KKKVHF
PF3D7_0407700^c^	conserved Plasmodium protein, unknown function	KKKVSW
PF3D7_0416900	conserved Plasmodium protein, unknown function	KRNVHF
PF3D7_0503000	50S ribosomal protein L28, apicoplast, putative	RKNVRF
PF3D7_0513600	deoxyribodipyrimidine photo-lyase, putative	KKQVSF
PF3D7_0526500	conserved Plasmodium protein, unknown function	KKCVHF
PF3D7_0526600	conserved Plasmodium protein, unknown function	KKKVTF
PF3D7_0610100^c^	pre-mRNA-splicing factor SLU7, putative (SLU7)	KKKVNF
PF3D7_0628100	HECT-domain (ubiquitin-transferase), putative	RKSVKF/KKSVTF
PF3D7_0713900	conserved Plasmodium protein, unknown function	KKNVNF
PF3D7_0717800	conserved Plasmodium protein, unknown function	KRSVSW
PF3D7_0723800	conserved Plasmodium protein, unknown function	KKSVQF
PF3D7_0727500	conserved Plasmodium protein, unknown function	KKCVSF
PF3D7_0804500	conserved Plasmodium membrane protein, unknown function	KKNVHF
PF3D7_0913600	conserved Plasmodium protein, unknown function	KKMVHF
PF3D7_0917500	conserved Plasmodium protein, unknown function	RKAVQF
PF3D7_0919900^c^	regulator of chromosome condensation, putative	KKSVSF
PF3D7_0922800	conserved Plasmodium protein, unknown function	KKNVCF
PF3D7_0930100	conserved Plasmodium protein, unknown function	KKTVHF
PF3D7_1002900	conserved Plasmodium protein, unknown function	KRNVNF
PF3D7_1003700	conserved Plasmodium protein, unknown function	KRKVKF
PF3D7_1008100^c^	conserved Plasmodium protein, unknown function	RKSVSF
PF3D7_1020600	conserved Plasmodium membrane protein, unknown function	KKKVNW
PF3D7_1022200	conserved Plasmodium membrane protein, unknown function	KKVVRF
PF3D7_1029400	conserved Plasmodium protein, unknown function	KKNVSF
PF3D7_1031600^c^	conserved Plasmodium protein, unknown function (GEXP15)	KKKVQF
PF3D7_1107700	pescadillo-like protein (PES)	KKKVTW
PF3D7_1106800	protein kinase, putative	KKKVSF
PF3D7_1109100	conserved Plasmodium protein, unknown function	RRKVSF
PF3D7_1113700	glyoxalase I (GloI)	KKNVKF
PF3D7_1128000.1	conserved Plasmodium protein, unknown function	KKNVTF
PF3D7_1139700	adrenodoxin reductase, putative	RKKVHF
PF3D7_1200100	erythrocyte membrane protein 1, PfEMP1 (VAR)	RKTVRF
PF3D7_1202600^c^	conserved protein, unknown function	KKNVTF/KKQVTF/KRTVSF/KKSVSF/KKTVSF/KKSVSF/KKNVSF
PF3D7_1220100	pre-mRNA splicing factor, putative	KKMVNF
PF3D7_1234700	CPW-WPC family protein	KKSVSF
PF3D7_1238500	conserved Plasmodium protein, unknown function	KKKVHF
PF3D7_1244200	transcription factor Tfb2, putative	RKSVHF
PF3D7_1244500	conserved Plasmodium protein, unknown function	RRKVNF
PF3D7_1322100	variant-silencing SET protein (SETvs)	KRNVSF
PF3D7_1364300	pre-mRNA-splicing factor ATP-dependent RNA helicase PRP16 (PRP16)	RKMVQF
PF3D7_1366300	conserved Plasmodium protein, unknown function	KKVVKF/KKKVQF
PF3D7_1367500	NADH-cytochrome b5 reductase, putative	KKHVHF
PF3D7_1406200	conserved Plasmodium protein, unknown function	KKMVSF
PF3D7_1411500	conserved Plasmodium protein, unknown function	KKQVSF/KKKVSF
PF3D7_1413000	conserved Plasmodium protein, unknown function	KKNVQF
PF3D7_1417300	cysteine protease ATG4, putative (ATG4)	KKKVRF
PF3D7_1460500^c^	conserved Plasmodium protein, unknown function	RKTVSF

^a^Annotations from PlasmoDB database (v24)

^b^The RVxF^ext^ binding motifs corresponding to the [KR][KR][ACHKMNQRSTV]V[CHKNQRST][FW] consensus sequence were indicated

^c^Shared proteins isolated by Y2H and *in silico* screenings

### Validation of interaction of Pips identified

All interactors identified by the above approaches could be capable of binding directly to PfPP1. To explore this further, we decided to investigate the physical interaction of PfPP1 with some of PfPips produced as recombinant proteins. To this end, a binding assay with purified His-tagged fusion Pips in *E. coli* and biotin labeled PfPP1 was used. This approach has already been used and validated using recombinant proteins of known partners of PfPP1 or derived peptides from binding motifs [17]. PfI2 and PfI3, previously described to interact with PfPP1, were used as positive controls. A total of 37 PfPips were successfully produced as recombinant proteins in *E. coli* and purified out of 68 candidates tested and corresponding to 66 different Pips.

**Fig. 2:**
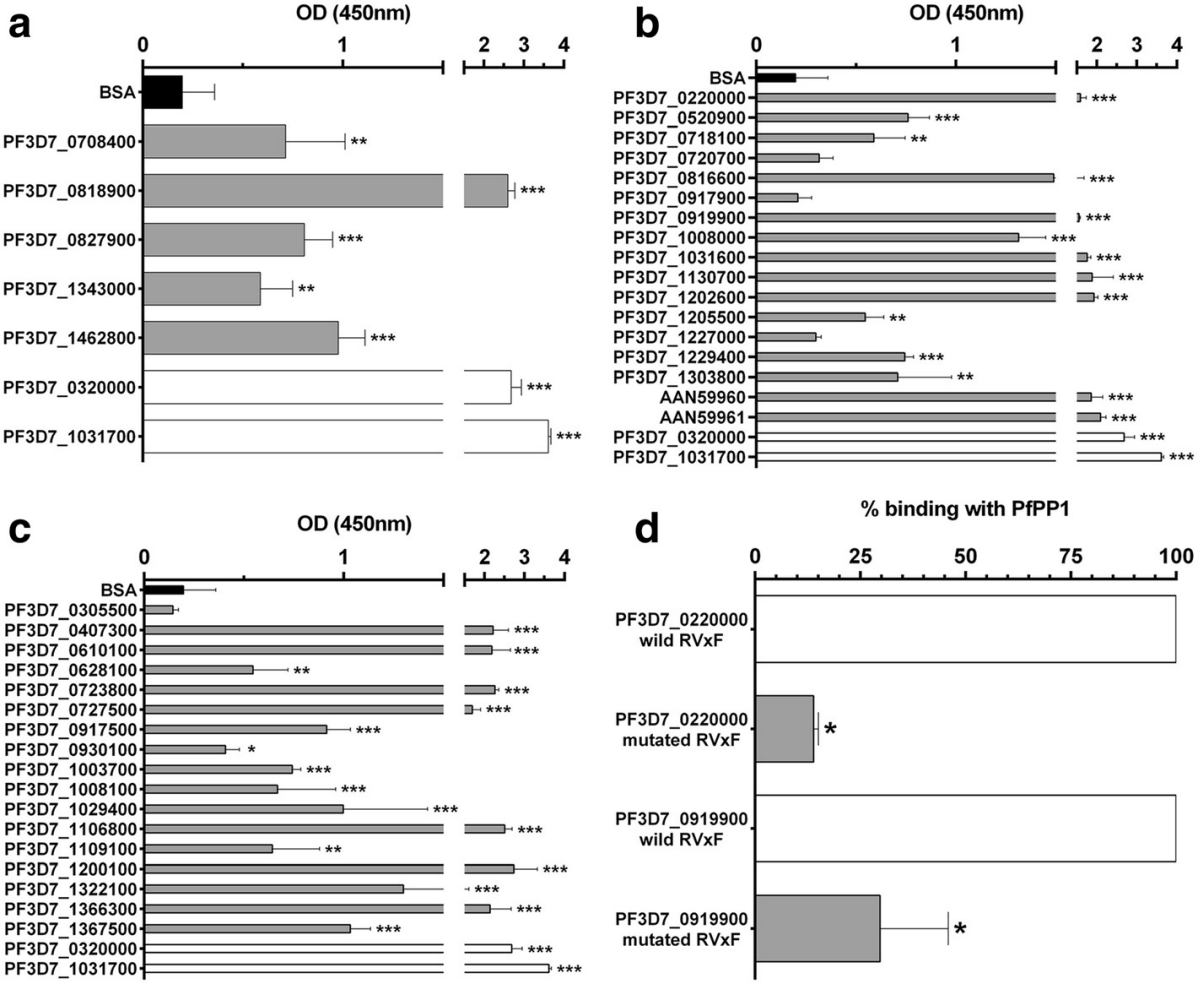
Direct interaction of Pips to PfPP1. The binding capacity of recombinant Pips (25pmol/well) to biotin-PfPP1 (4pmol/well) was measured using an ELISA-based assay. **a** represents the binding of recombinant Pips identified by PP1-affinity column (*gray bars*). PfI2 (PF3D7_0320000) and PfI3 (PF3D7_1031700) were used as positive controls (*white bars*) and BSA was used as negative control (*black bars*). **b** represents the binding of Pips identified by Y2H screening (*gray bars*). AAN59960 and AAN59961 are human H2A and H2B proteins respectively which present high identity with PfH2A (PF3D7_0617800) and PfH2B (PF3D7_1105100). **c** represents the Pips identified by genome *in silico* analysis (*gray bars*). **d** contribution of the RVxF motifs to the binding of PF3D7_0220000 and PF3D7_0919900 to PfPP1 (*gray bars*). The interaction of PfPP1 to wild proteins was considered at 100 % binding. Results are presented as mean ± SD of 2 independent experiments preformed in duplicate. *p < 0.05, **p < 0.01 and ***p < 0.001 (Mann–Whitney *U* test), compared to the negative control

In the case of the Pips identified by PfPP1 affinity column, 5 proteins were produced out of 6. Binding data presented in Fig. 2a showed that biotin-PfPP1 binds to the 5 proteins tested. These data likely suggests that the identified Pips are direct interactors of PfPP1.

Next, we searched to check the direct interactions of Pips identified by Y2H screening and PfPP1. Fifteen recombinant PfPips were produced out of 30 cloned cDNAs tested (Additional file 5: Figure S1, Additional file 6: Table S5). The failure for the production of some proteins could be due to the lack of expression and/or to the toxicity for bacteria. For the binding with H2A and H2B, commercially available human recombinant proteins were used as they showed 67 and 63 % identity with PfH2A and PfH2B respectively (Additional file 7: Figure S2). As depicted in Fig. 2b, biotin-PfPP1 was able to bind to 14 recombinant proteins out of 17 tested. These data confirmed a direct interaction between PfPP1 and these Pips. Moreover, this binding assay showed that Pips with or without the RVxF motif can bind PfPP1 and confirmed that proteins which are out of frame of GAL4AD could be true Pips. However, under the same conditions, 3 proteins did not show significant binding when compared to BSA. The lack of binding is unlikely to be due to an absence of coating of purified protein to wells as the quality of coating was checked using an anti-His antibody (data not shown). It could be argued that these proteins are not direct partners of PfPP1 or their interaction may require additional partner(s) expressed by the yeast.

Finally, in order to examine the binding capacity of several *in silico* Pips identified by genome analysis, 32 fragments which correspond to 30 potential candidates were tested for protein production. The fragments were about 190 residues long with the RVxF^ext^ motif in the middle of the recombinant proteins (Additional file 5: Figure S1, Additional file 6: Table S5). The expression and purification were successful for only 17 proteins. Their ability to bind PP1 was then examined as described above. Significant interactions for 16 out of 17 proteins tested were observed (Fig. 2c). The lack of interaction of PfPP1 with PF3D7_0305500 could be attributed to the fact that its RVxF is not a valid binding motif. However, it is important to point out that 6 Pips identified by Y2H and *in silico* screens (PF3D7_0220000, PF3D7_0610100, PF3D7_0919900, PF3D7_1008100, PF3D7_1031600 and PF3D7_1202600) were confirmed to interact with PfPP1 by ELISA (Fig. 2b and c), highlighting the accuracy of the RVxF consensus sequence used throughout this study.

To further explore the implication of the RVxF motifs in PfPP1 binding, 2 Pips identified by Y2H screening and *in silico* analysis were mutated in their RVxF motifs and the corresponding recombinant proteins were produced. Results presented in Fig. 2d showed a significant decrease of the binding with PfPP1 (about 86 % for PF3D7_0220000 and 70 % for PF3D7_0919900). This confirms that the RVxF motifs of these 2 Pips are the main contributors for binding to PfPP1. However, it could not be excluded that these interactions could involve secondary structures of the protein to stabilize and to lead to a functional complex. This could be attributed to the fact that the RVxF binding motif, in most regulators, is often present in intrinsically disordered regions and the protein could adopt a structure upon binding [16, 17].

The newly RVxF-containing PfPips were then used to reexamine the features of the RVxF motif and the nature of the flanking amino acids in *P. falciparum*. The accepted motif exhibits the sequence [K_52_R_13_][K_55_R_10_][K_19_N_15_S_13_T_5_]V[S_22_H_11_T_9_R_6_Q_6_N_6_][F_60_W_5_] (Additional file 8: Figure S3). Interestingly, the amino acids between V and F/W are enriched by S and H (~50 %). At the N-terminal positions of the RVxF (−1, and −2), the most conserved amino acid is the K residue. We have also found a high frequency of L residue at −2. Based on these data, the RVxF consensus sequence for PfPips could be extended and refined as [K_13_L_12_I_7_V_6_][K_18_N_8_I_8_R_5_][K_52_R_13_][K_55_R_10_][K_19_N_15_S_13_T_5_]V[S_22_H_11_T_9_R_6_Q_6_N_6_][F_60_W_5_].

### PfPP1 interaction networks

**Fig. 3:**
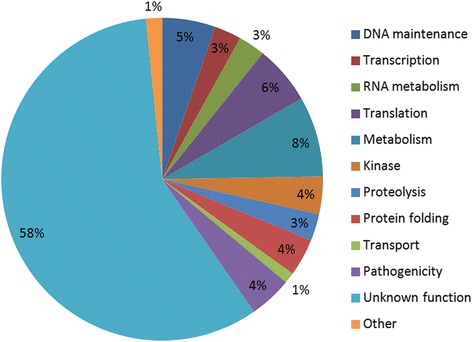
Pie chart of biological functions of Pips. A total of 186 proteins are classified in 12 different biological functions based on confirmed or putative functions indicated in PlasmoDB and shown in our pie chart

The identification in this study of 186 new Pips provides new insights on the functional diversity of PfPP1 functions. Among the 186 identified proteins, 108 (58 %) have unknown function. For the 78 remaining proteins, the most abundant proteins correspond to metabolism, DNA maintenance, translation and phosphorylation processes (Fig. 3).

**Fig. 4:**
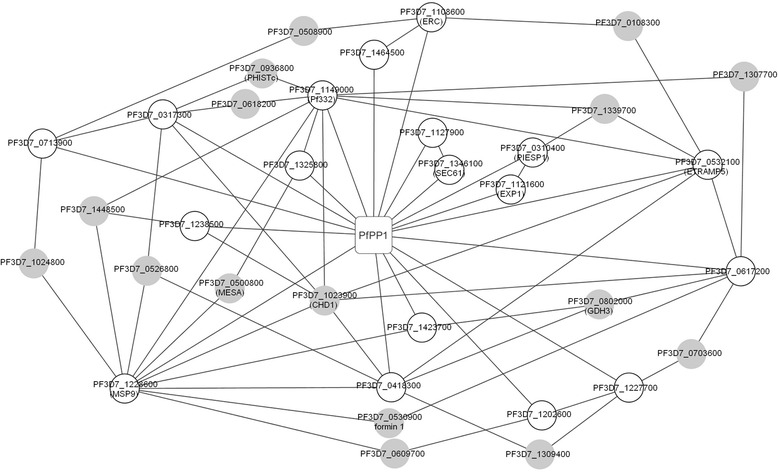
PfPP1 Pathogenicity network. The graph shows interactions (*lines*) between Pips identified by our different approaches (*white circles*) and PfPP1 (*square box*). The gray circles represent connecting proteins based on the results of Lacount et al. [34]. When available, gene names given in PlasmoDB are shown in brackets

Based on an earlier published *P. falciparum* interactome [34] and on the identified PP1 interacting proteins reported in this work, we were able to establish three networks where several Pips could be involved. The transcription and DNA maintenance network illustrated in Figure S4A (Additional file 9) contained 9 direct Pips (white circles) detected by the different approaches described above and 4 connecting proteins (gray circles). The folding/proteolysis network involved 8 direct Pips and 2 connecting proteins (Additional file 9: Figure S4B). The pathogenicity network comprised 18 direct Pips and 16 connecting proteins (Fig. 4). In the latter network, 2 homologs of direct Pips in *P. berghei* (PBANKA_0408500 homolog to PF3D7_0310400 and PBANKA_0926700 homolog to PF3D7_1121600) have been shown to be essential for parasite survival as no viable KO parasites can be obtained [35, 36]. It is worthy of note that PF3D7_1023900, a connecting protein, contains a region of about 700 amino acids with 40 % identity with a yeast protein (YER164W) which has been shown to interact with yeast PP1 [23]. Its homolog in *P. berghei* (PBANKA_050810) has been shown to be essential for parasite survival [35]. The network analysis raises the possibility that new Pips could play a role in pathogenicity, at least in part, through their interaction with and regulation of PfPP1. However, it cannot be excluded that these proteins could be also substrates of PfPP1.

## Conclusion

The diversity of proteins identified by three different and complementary approaches suggests that PP1c has a wide interaction network which controls several aspects of parasite physiology. A total of 186 proteins were identified by these approaches. One protein was common to both Y2H screening and PfPP1-affinity column approach and 8 proteins to Y2H and *in silico* screenings. This provides experimental support to determine the contribution of the RVxF motif to PfPP1 binding when present in Pips, to identify new binding regions/motifs specific of *Plasmodium* and to explore the role of Pips as regulators, substrates or both. Further experiments need to be done to determine PfPP1 substrates, for instance by identifying the phosphoproteomes of Pf in vitro in the absence and presence of recombinant active PfPP1, and to evaluate whether the new Pips could be able to regulate PfPP1 activity. These future studies will help to better elucidate PP1 signaling in *Plasmodium* and to increase and improve the choice of parasite targets for drug design.

## Methods

### Affinity purification of Pips

For affinity purification, 10 mg of PfPP1 recombinant protein (produced as previously described [17]) were covalently coupled to 0.5 ml of CNBr-activated Sepharose 4B according to the supplier’s instructions (Sigma). The coupling efficiency was > 90 %. Before use, PP1 beads were submitted to 3 wash cycles with 3 M NaSCN and PBS. Ten mg of total soluble parasite extract (prepared as previously described [17]) were precleared on 2 ml of activated sepharose beads blocked with ethanolamine. The precleared extract recovered by centrifugation was filtered, divided in 2 equal volumes and incubated with BSA-sepharose beads or PP1-beads overnight at 4 °C. After 5 washings with 50 mM Tris, 0.3 M NaCl, proteins were eluted with 2 × 0.5 ml of 3 M NaSCN. The proteins were then precipitated with acetone and separated on SDS-PAGE. No stained bands were observed for eluates of BSA-sepharose beads. Stained bands observed for eluates of PP1 column affinity were cut for MS/MS identification as previously described [37].

### Plasmid and yeast strain

The cDNA library of *Plasmodium falciparum*, cloned in pGAD-HA vector, was purchased from Dualsystems Biotech. In this plasmid, the expressed parasite proteins are fused to the GAL4 activation domain. Yeast Y2HGold strain (Clontech) was transformed with the pGAD-cDNA library according to the method for high-efficiency library scale transformation of Dualsystems Biotech, and allowed to form colonies on 150 mm plates with SD agar medium lacking leucine (SD/-Leu) at 30 °C (3-4 days). Y2HGold contains four integrated reporter genes (AUR1-C, HIS3, ADE2 and MEL1) under the control of three distinct GAL4 responsive promoters used to detect two-hybrid interaction and in order to reduce false positives. All clones were scraped, harvested with a freezing medium (YPDA medium with 25 % glycerol) and stored in 1 ml aliquots at −80 °C for further experiments. This library-transformed yeast strain is referred below as the prey strain.

The cDNA encoding for *P. falciparum* protein phosphatase type-1 (PfPP1, PlasmoDB accession number: PF3D7_1414400) was cloned in the pGBKT7 vector (Clontech) to generate a PfPP1-pGBKT7 plasmid allowing the production of PfPP1 fused with the GAL4 DNA binding domain. Y187 yeast competent cells (Clontech) were then transformed with this construct according to the manual of Yeastmaker™ Yeast Transformation System 2 (Clontech). The expression of PfPP1 was checked by immunoblot using anti-GAL4BD antibody. This strain was used as a bait to screen protein interactions with the *P. falciparum* cDNA library by mating.

### Yeast two-hybrid assay

All reagents and methods for yeast-two hybrid assays were from the Matchmaker™ Gold Yeast Two-Hybrid System (Clontech).

One fresh and large colony of bait yeast strain (pGBKT7-PfPP1 in Y187) was inoculated into 50 ml of SD/-Trp/Kan (Kanamycin) liquid medium agitated at 250 rpm, 30 °C until the OD600 reached 0.8. The supernatant was discarded after centrifugation for 5 min at 1,000 g. The pellet was suspended in 5 ml of SD/-Trp/Kan liquid medium ([cells] >1 × 10^8^/ml). 1 ml of library prey strain (after rechecking the titer) and 5 ml of bait strain Y187 were combined with 45 ml of 2xYPDA liquid medium and incubated for 20–24 h at 30 °C at 40 rpm. The culture was centrifuged for 10 min at 1,000 g, then 50 ml of 0.5xYPDA liquid medium (with 50 μg/ml Kan) was added to resuspend the pellet. After a centrifugation for 10 min, cells were suspended in 10 ml of 0.5xYPDA liquid medium (with 50 μg/ml Kan) and the total volume was measured. To calculate the number of screened clones and the mating efficiency, 100 μl of the fusion culture (diluted by 10^1^ gradient) were plated on the selective medium SD/-Trp, SD/-Leu, and SD/-Trp/-Leu (Double dropout medium, DDO) and incubated for 3–5 days at 30 °C. More than 1 million diploids were screened to maximize chances of detecting genuine interactions on Aureobasidin A (AbA) plates. The remaining fusion culture was spread onto selective medium SD/-Trp/-Leu/Kan supplemented with 125 ng/ml AbA (DDO/A), 200 μl per 150 mm plates and incubated for 3–7 days at 30 °C. Colonies obtained were re-streaked onto higher stringency SD/-Trp/-Leu/-His/AbA/Kan (TDO/A, Triple dropout supplemented with AbA) and SD/-Trp/-Leu/-His/-Ade/AbA/Kan (QDO/A, Quadruple dropout supplemented with AbA) agar plates. This screening was carried out five times independently.

### Identification of potential Pips

The prey plasmids from positive yeast clones were isolated using the Plasmid DNA Purification kit (Macherey-Nagel). To facilitate yeast lysis, the equivalent of 100 μl of glass beads (Sigma) was added in the resuspension buffer. To amplify the plasmid obtained, it was transformed to *E. coli* DH5α cells (Life Technologies) followed by selection on LB/Ampicillin and LB/Kanamycin plates. Plasmids were isolated once again using the Plasmid DNA Purification kit and then to estimate the sizes of the specific inserts on positive prey plasmids, plasmids were digested using the *Sfi*I restriction enzyme.

The positive prey plasmids were characterized by sequencing and BLAST searches to identify the corresponding *P. falciparum* genes in the PlasmoDB database (v24).

### *In silico* screening

The consensus sequence used to identify potential interactors of PfPP1, was [KR][KR][ACHKMNQRSTV]V[CHKNQRST][FW]. It was based on the crystal of PP1 with a RRVSFA peptide [38] and on consensus sequences defined by different publications [12, 30, 31]. After identification of the putative partners, the positions −10 to +10 of RVxF motifs were aligned by BioEdit software (v7.2.5) to establish a new consensus sequence.

### Recombinant proteins expression and purification

In order to verify the interaction between PfPP1 and its new interactors, His-tagged recombinant proteins were produced. Concerning Pips from Y2H screening, the recombinant proteins correspond to the fragment identified during the screening (Additional file 6: Table S5). For Pips from *in silico* screening, the A/T rich regions or acidic amino acids were eliminatory to avoid a lack of expression in *E.coli*. The different RVxF motifs were placed in the middle of recombinant proteins. The chosen coding regions for Pips were obtained by PCR with the primers mentioned in Additional file 10: Table S6 using the Advantage 2 Polymerase Mix (Clontech). The PCR conditions consisted of 10 min at 94 °C followed by 35 cycles at 94 °C (45 s), 56 °C or 58 °C (1 min) and 68 °C (1 min 30), finished by 7 min at 68 °C. To examine the role of RVxF binding motif, mutated constructs (KSVSF to KSASA for PF3D7_0919900, and the introduction of a stop in the second position of the KKVRF motif for PF3D7_0220000) were obtained by a PCR-based site-directed mutagenesis approach using the wild pQE30-PF3D7_0919900 and pETDuet-PF3D7_0220000 constructions as templates, the primers mentioned in Additional file 10: Table S6 and Isis DNA polymerase (MP Biomedicals). The mutations were checked by sequencing.

For the expression of Pips, the pETDuet-1 (Novagen) or pQE30 (Qiagen) expression system and the In-Fusion HD Cloning system (Clontech) were used according to the supplier’s instructions. The plasmids and restriction sites are mentioned in Additional file 10: Table S6. The constructions obtained were checked by sequencing and transformed in One Shot® BL21 Star™ (DE3) Chemically Competent *E. coli* cells (Life Technologies) for expression.

The expression of His6-Pips was carried out in the presence of 0.5 mM IPTG at 37 °C for 2 h. Cells were harvested in sonication buffer (20 mM Tris, 500 mM NaCl, 6 M Guanidine, 20 mM Imidazole and protease inhibitor cocktail (Roche), pH 7.5). Recombinant proteins were purified according to manufacturer’s instructions by Ni^2+^-NTA agarose beads (Macherey Nagel). Washing steps were performed with a buffer containing 20 mM Tris, 500 mM NaCl and 20 mM imidazole, pH 7.5. Elution was done with a buffer containing 20 mM Tris, 500 mM NaCl and 600 mM imidazole, pH 7.5. The eluted proteins were dialyzed against 20 mM Tris, 500 mM NaCl, pH 7.5.

PfPP1 was produced as described above [16] with a dialysis buffer containing 20 mM Tris, 500 mM Nacl and 1 mM MnCl_2_, pH 7.5.

The produced proteins were quantified with the Pierce™ BCA Protein Assay Kit (Life Technologies) and checked by western blot. Soluble proteins were separated on a 4–20 % SDS-PAGE and subsequently blotted onto nitrocellulose. The blots were probed with anti-His antibody (1:2000 dilution) (Qiagen). As secondary antibody, a horseradish peroxidase-labeled anti-mouse IgG (1:20000 dilution) was used, followed by chemiluminescence detection with SuperSignal™ West Dura Extended Duration Substrate (Life Technologies).

### Measurement of Pips binding

Binding of Pips to PfPP1 was assessed by an ELISA-based assay as previously described [17]. The plates were coated with 25pmol of each Pips in PBS overnight at 4 °C. In these experiments, recombinant PfI2 and PfI3 [16, 17] and BSA were used as positive and negative controls respectively. The statistical significance was calculated with the Mann-Whitney *U* test for nonparametric data and *p* < 0.05 was considered significant.

### Bioinformatic analysis

The different binding motifs were identified using the tool “Protein Motif Pattern” available on PlasmoDB website. For biological functions of Pips, only the putative or characterized functions were taken into account. The GO annotation of unknown proteins was not retained. For functional analysis, the different protein subnetworks were based on the results from LaCount [34] and our interactions with PfPP1. After manual correction, they were visualized on Cytoscape (v3.2.1). The Venn diagram was established from Venny [39].

### Availability of data and materials

All genome and protein sequence files are available through PlasmoDB (http://plasmodb.org/plasmo/) and GenBank (http://www.ncbi.nlm.nih.gov/protein/) and the data are included in the manuscript and in Additional file 1: Table S1, Additional file 2: Table S2, Additional file 3: Table S3, Additional file 4: Table S4, Additional file 6: Table S5, Additional file 7: Figure S2, Additional file 8: Figure S3, and Additional file 9: Figure S4.

## Additional files

Additional file 1: Table S1. Pips identified by affinity chromatography/mass spectrometry. (XLSX 10 kb) Additional file 2: Table S2. Common Pips between *P. falciparum* and other species. (XLSX 13 kb) Additional file 3: Table S3. Pips identified by Yeast two-hybrid screening. (XLSX 24 kb) Additional file 4: Table S4. Pips identified by genome *in silico* analysis. (XLSX 13 kb) Additional file 5: Figure S1. Production of Pips in *E. coli*. Immunoblot assay of recombinant Pips. (PDF 143 kb) Additional file 6: Table S5. Recombinant proteins produced. (XLSX 15 kb) Additional file 7: Figure S2. Conservation of human and Pf H2A and H2B. Global alignments using BioEdit software. (DOCX 58 kb) Additional file 8: Figure S3. Global alignment between *in silico* Pips. The different protein sequences flanking the RVXF motifs were aligned using BioEdit software. (DOCX 463 kb) Additional file 9: Figure S4. PfPP1 interaction networks. Transcription/DNA maintenance and Folding/Proteolysis subnetworks are shown. (PNG 141 kb) Additional file 10: Table S6. List of primers for recombinant proteins expression. (DOCX 30 kb)

## Abbreviations

AbA: Aureobasidin A
DDO: Double dropout medium
GAL4AD: GAL4 activation domain
HSP: Heat shock protein
I2: Inhibitor 2
I3: Inhibitor 3
Kan: Kanamycin
KO: Knock out
LRR1: Leucine Rich Repeat 1
Pf: *Plasmodium falciparum*
Pips: PP1 interacting proteins
PP1: Protein Phosphatase 1
TDO: Triple dropout medium
Y2H: Yeast two-hybrid
