# Supplementary material for: Analysis of the interactome of the Ser/Thr Protein Phosphatase type 1 in Plasmodium falciparum
Source: BMC Genomics. 2016 Mar 17;17:246. doi: 10.1186/s12864-016-2571-z (PMC4794898; doi:10.1186/s12864-016-2571-z)
Supplement: Additional file 5: Figure S1. — Production of Pips in E. coli. Immunoblot assay of recombinant Pips. (PDF 143 kb) [file 12864_2016_2571_MOESM5_ESM.pdf]

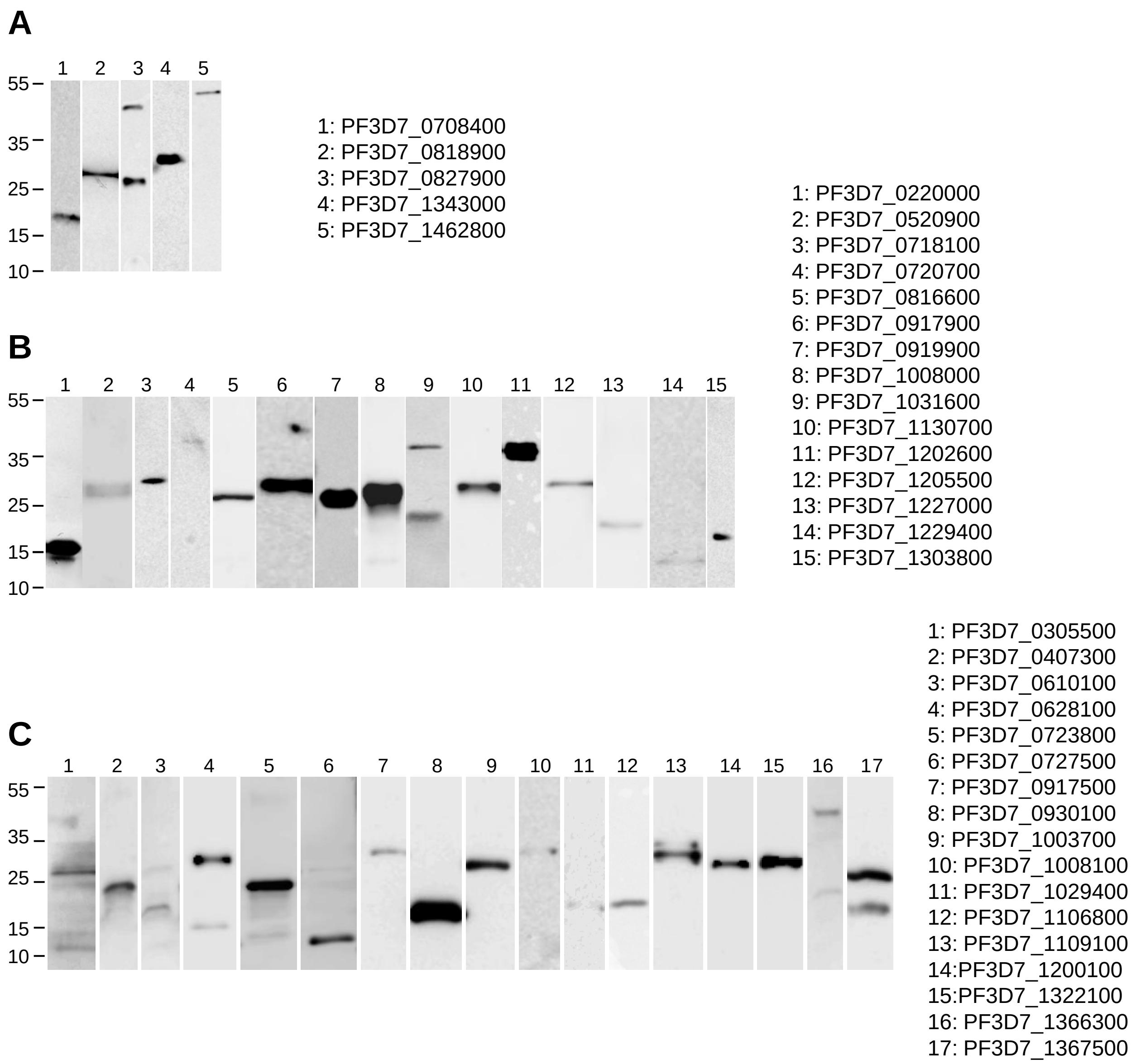

Figure S1. Production of Pips in *E. coli*. Purified recombinant proteins were separated by SDS-PAGE and transferred to nitrocellulose membrane. Immunoblot assays were performed using an anti-histidine mAb at 1:2000. **A**, **B** and **C**, represent Pips identified by affinity purification, Y2H screening and *in silico* analysis respectively.
