# Supplementary material for: Analysis of the interactome of the Ser/Thr Protein Phosphatase type 1 in Plasmodium falciparum
Source: BMC Genomics. 2016 Mar 17;17:246. doi: 10.1186/s12864-016-2571-z (PMC4794898; doi:10.1186/s12864-016-2571-z)

**Figure S2. Conservation of human and Pf H2A and H2B.** Global alignments of human H2A (AAN59960) with PfH2A (PF3D7_0617800) (**A**) and human H2B (AAN59961) with PfH2B (PF3D7_1105100) (**B**) showed 67% and 63% of identity respectively.

**A**


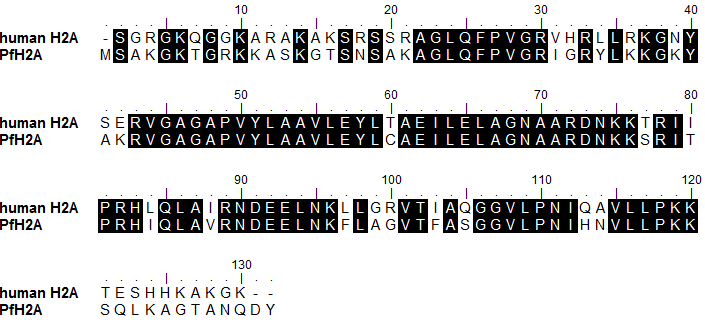


**B**


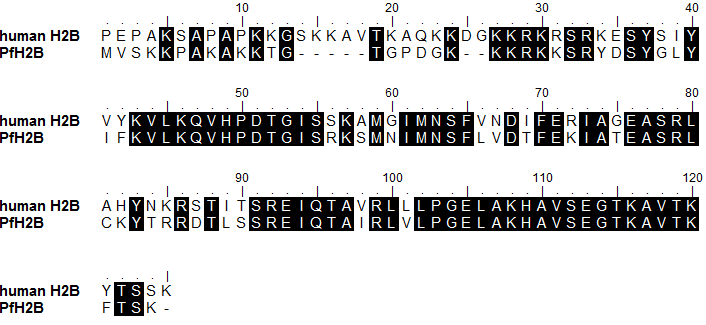

Supplement: Additional file 7: Figure S2. — Conservation of human and Pf H2A and H2B. Global alignments using BioEdit software. (DOCX 58 kb) [file 12864_2016_2571_MOESM7_ESM.docx]
