# Supplementary material for: Analysis of the interactome of the Ser/Thr Protein Phosphatase type 1 in Plasmodium falciparum
Source: BMC Genomics. 2016 Mar 17;17:246. doi: 10.1186/s12864-016-2571-z (PMC4794898; doi:10.1186/s12864-016-2571-z)
Supplement: Additional file 8: Figure S3. — Global alignment between in silico Pips. The different protein sequences flanking the RVXF motifs were aligned using BioEdit software. (DOCX 463 kb) [file 12864_2016_2571_MOESM8_ESM.docx]

**
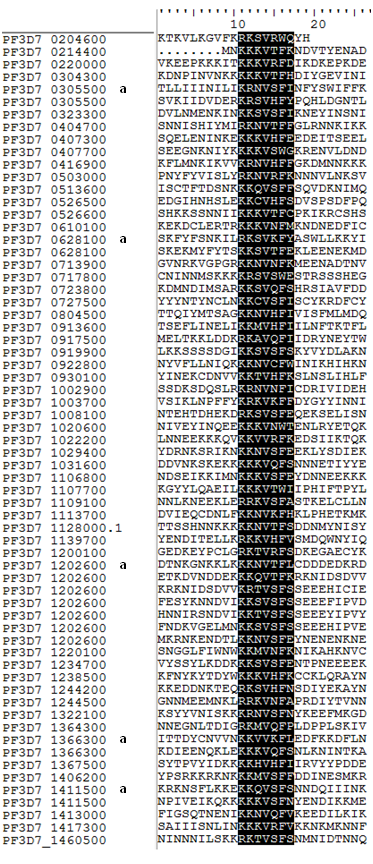
Figure S3. Global alignment between *in silico* Pips.** The different protein sequences flanking the RVxF^ext^ motifs (black) were aligned using BioEdit software. The PlasmoDB accession numbers are indicated.

**a**, proteins containing at least 2 RVxF^ext^ motifs.
