# Supplementary figures and images for: Analysis of the interactome of the Ser/Thr Protein Phosphatase type 1 in Plasmodium falciparum
Source: BMC Genomics. 2016 Mar 17;17:246. doi: 10.1186/s12864-016-2571-z (PMC4794898; doi:10.1186/s12864-016-2571-z)

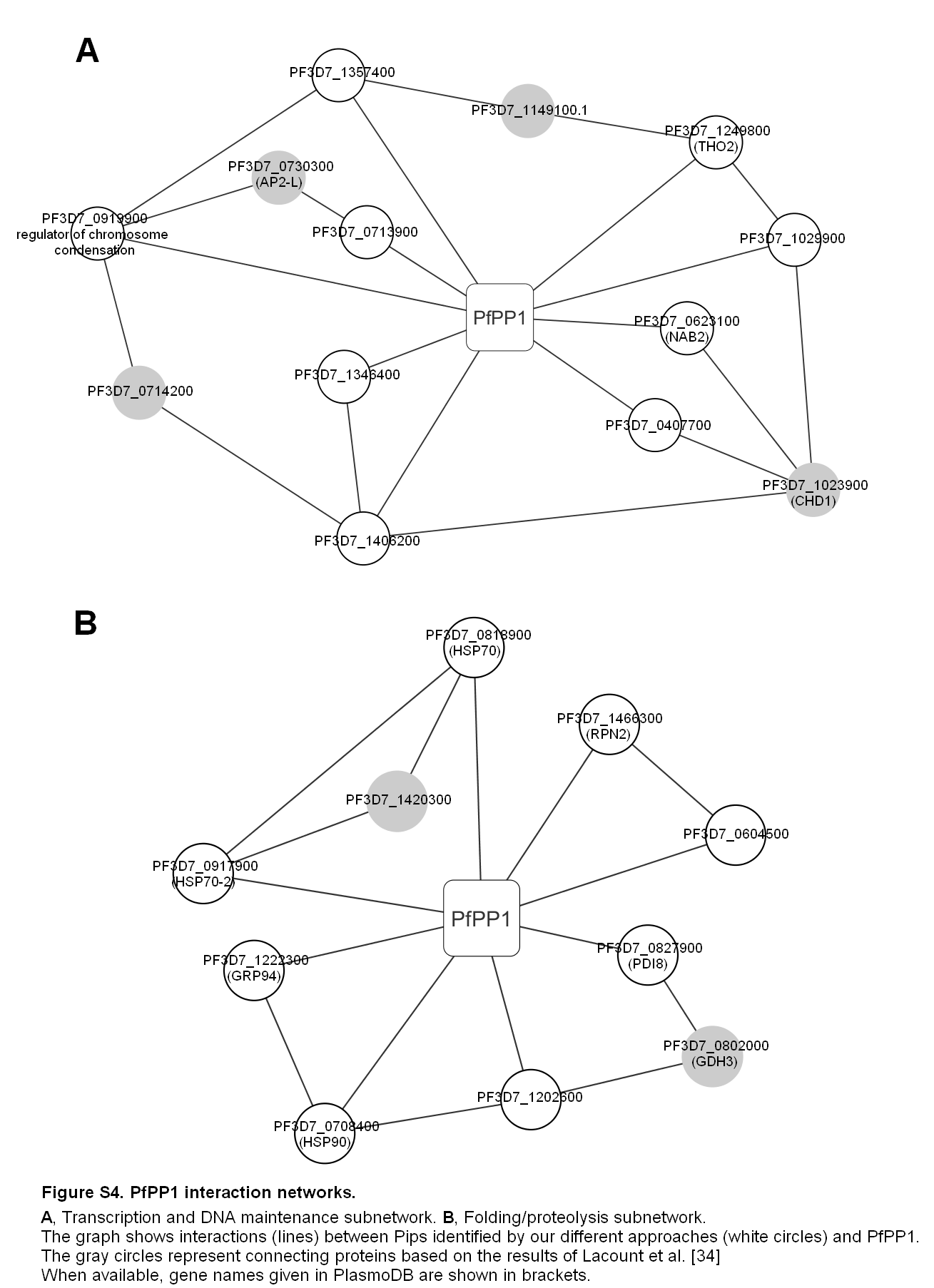

Supplement: Additional file 9: Figure S4. — PfPP1 interaction networks. Transcription/DNA maintenance and Folding/Proteolysis subnetworks are shown. (PNG 141 kb) [file 12864_2016_2571_MOESM9_ESM.png]
