# Supplementary material for: Analysis of the interactome of the Ser/Thr Protein Phosphatase type 1 in Plasmodium falciparum
Source: BMC Genomics. 2016 Mar 17;17:246. doi: 10.1186/s12864-016-2571-z (PMC4794898; doi:10.1186/s12864-016-2571-z)
Supplement: Additional file 10: Table S6. — List of primers for recombinant proteins expression. (DOCX 30 kb) [file 12864_2016_2571_MOESM10_ESM.docx]

**Table S6. List of primers for recombinant proteins expression.**

|  | Accession number | Primer Orientation | Sequence | Plasmid | Restriction enzyme sites |
| --- | --- | --- | --- | --- | --- |
|  |  |  |  |  |  |
| Affinity purification | PF3D7_0608800  PF3D7_0708400  PF3D7_0818900  PF3D7_0827900  PF3D7_1343000  PF3D7_1462800 | F  R  F  R  F  R  F  R  F  R  F  R | CATCACCACAGCCAGGATCCCGTTAAAGAATTAAAAAGTAGTC  ACTTAAGCATTATGCGGCCGCCAATAATTTCAGTACATTCATC  CATCACCACAGCCAGGATCCCACAGTTGAACATGAATGG  ACTTAAGCATTATGCGGCCGCCCTCGTGGATACCCAAC  CACAGCCAGGATCCGAATTCACAAGCAGCCATTTTATCTG  ACTTAAGCATTATGCGGCCGCTTTCATACATGTTTCAATTTCAG  CATCACCACAGCCAGGATCCGGGTCATTGTAAAAGGCTAATC  ACTTAAGCATTATGCGGCCGCGCCGTCTTCAAATTCAGG  CATCACCACAGCCAGGATCCGGCTTTGATTGAAAACTTAAACTC  ACTTAAGCATTATGCGGCCGCGGTGGCCTTAAAATAACC  CATCACCACAGCCAGGATCCGGCAGTAACAAAACTTGG  ACTTAAGCATTATGCGGCCGCGTTGTTAGTAATGTGTAC | pETDuet-1  pETDuet-1  pETDuet-1  pETDuet-1  pETDuet-1  pETDuet-1 | BamHI-NotI  BamHI-NotI  EcoRI-NotI  BamHI-NotI  BamHI-NotI  BamHI-NotI |
|  |  |  |  |  |  |
| Y2H screening | mal_mito_2  PF3D7_0107600  PF3D7_0207500 | F  R  F  R  F  R | CACAGCCAGGATCCGAATTCACTATATAATATGATATTTACAATT  ACTTAAGCATTATGCGGCCGCAGTATCAACTTCTAAACCAGTA  CATCACCACAGCCAGGATCCGGGGTTAATGTATTATTTCAC  ACTTAAGCATTATGCGGCCGCGTATAATCATATGATGCTAAC  CACAGCCAGGATCCGAATTCAATGATATGTCCTATTTTTTTCC  ACTTAAGCATTATGCGGCCGCCCCATTATAATTTTTTAAAAGTCC | pETDuet-1  pETDuet-1  pETDuet-1 | EcoRI-Not  BamHI-NotI  EcoRI-NotI |
|  | PF3D7_0220000 | F  R | CATCACCACAGCCAGGATCCAGGTTTGGAAGAAGATGATTTAGAAGAAG  CATTATGCGGCCGCAAGCTTGGTTCCTTATCCTTAATATCAAACC | pETDuet-1 | BamHI-HindIII |
|  | PF3D7_0418300  PF3D7_0520800 | F  R  F  R | CATCACCACAGCCAGGATCCGGGATCTGGTGGACCCTG  ACTTAAGCATTATGCGGCCGCCCCCTTGTTCATAATCC  CACAGCCAGGATCCGAATTCAGAATATGATTTGACATTTGTGC  ACTTAAGCATTATGCGGCCGCTATGGTTTCACTATCACTATTTC | pETDuet-1  pETDuet-1 | BamHI-NotI  EcoRI-NotI |
|  | PF3D7_0520900 | F  R | CACAGCCAGGATCCGAATTCAGTGTATGGATGTAGACATTC  ACTTAAGCATTATGCGGCCGCGTATCTGTATTCGTTACTC | pETDuet-1 | EcoRI-NotI |
|  | PF3D7_0623100  PF3D7_0718100  PF3D7_0720700  PF3D7_0724600  PF3D7_0814000 | F  R  F  R  F  R  F  R  F  R | CACAGCCAGGATCCGAATTCAAACGCTAAAAGTAGCAATG  ACTTAAGCATTATGCGGCCGCGCTATCACTAATTCTTTTATATGTT  CATCACCACAGCCAGGATCCGAAAATAGGAGAACAAATTGC  ACTTAAGCATTATGCGGCCGCGTTTCTTTTGTTATCTCTTC  CATCACCACAGCCAGGATCCGGATGATGAATACGATAATGG  ACTTAAGCATTATGCGGCCGCGTTAATTATAATGTTACTATCC  CACAGCCAGGATCCGAATTCAATGTATGTTTATAACAAACAAAATA  ACTTAAGCATTATGCGGCCGCTAATATTTTAATATTATTCAAATTATAG  CATCACCACAGCCAGGATCCGAAAAAAAAAAAAAGAAGATTGTTGA  ACTTAAGCATTATGCGGCCGCAGCCAATTTAGCTCTACGTAA | pETDuet-1  pETDuet-1  pETDuet-1  pETDuet-1  pETDuet-1 | EcoRI-NotI  BamHI-NotI  BamHI-NotI  EcoRI-NotI  BamHI-NotI |
|  | PF3D7_0816600 | F  R | CACAGCCAGGATCCGAATTCAGATGCAGTACGTAAAAAACCAT  ACTTAAGCATTATGCGGCCGCACTTTTTGAAAAGTGCAACTTC | pETDuet-1 | EcoRI-NotI |
|  | PF3D7_0917900 | F  R | CATCACCACAGCCAGGATCCGGATGAAATTGTTTTAGTAGGAG  ACTTAAGCATTATGCGGCCGCGATTTTATCAGCTAATTTATCTTTA | pETDuet-1 | BamHI-NotI |
|  | PF3D7_0919900 | F  R | ACCATCACCATCACGGATCCACAAATAACGAATATGAAGATGAAGAAAG  AAGCTCAGCTAATTAAGCTTAGTTCTTCCTTTAAAGTGTTATTTTTATTC | pQE30 | BamHI-HindIII |
|  | PF3D7_1008000 | F  R | CACAGCCAGGATCCGAATTCAGATTCTGCTATATCTAATAAATTAAG  ACTTAAGCATTATGCGGCCGCATCAAATCGATGTATGGAGAAACAA | pETDuet-1 | EcoRI-NotI |
|  | PF3D7_1023400 | F  R | CACAGCCAGGATCCGAATTCATTTATTGAGGCTTTTACTCATC  ACTTAAGCATTATGCGGCCGCCGTGACATTTTTTGGACTTTC | pETDuet-1 | EcoRI-NotI |
|  | PF3D7_1031600 | F  R | CATCACCACAGCCAGGATCCAGGGGAGGAAAATGACTTGGATAAATTC  CATTATGCGGCCGCAAGCTTGTTAATTCCATTCAATATGGTATTTTTAT | pETDuet-1 | BamHI-HindIII |
|  | PF3D7_1130700 | F  R | CACAGCCAGGATCCGAATTCAGGTCTTTATAAAGAAAATGATTTG  ACTTAAGCATTATGCGGCCGCTTTTTTCTTTTCATTTAAATAATTC | pETDuet-1 | EcoRI-NotI |
|  | PF3D7_1202600 | F  R | CATCACCACAGCCAGGATCCAGATCTAGATGATGAAAAGAAGAAACAGGTAAC  CATTATGCGGCCGCAAGCTTCTCGAGTTCTTGCTCAACCTCCTTTAATAATTTAC | pETDuet-1 | BamHI-HindIII |
|  | PF3D7_1205500 | F  R | CACAGCCAGGATCCGAATTCAGTAGTAGATTGTGGTTCTAC  ACTTAAGCATTATGCGGCCGCGTTTGTATTATGCAATGATATGG | pETDuet-1 | EcoRI-NotI |
|  | PF3D7_1227000  PF3D7_1228600 | F  R  F  R | CACAGCCAGGATCCGAATTCAATGATGGAACTTGAAGAACAAC  ACTTAAGCATTATGCGGCCGCAACATTGTCGTCTTTTTTTTTTAG  CATCACCACAGCCAGGATCCGGCTCATAAGAAATTAATTAAAGC  ACTTAAGCATTATGCGGCCGCGATACCCATATCTAGAATAC | pETDuet-1  pETDuet-1 | EcoRI-NotI  BamHI-NotI |
|  | PF3D7_1229400 | F  R | CACAGCCAGGATCCGAATTCAATGCCTTGCTGTGAAGTAATA  ACTTAAGCATTATGCGGCCGCGCCGAAAAGAGAACCACTG | pETDuet-1 | EcoRI-NotI |
|  | PF3D7_1303800  PF3D7_1308400  PF3D7_1325800  PF3D7_1346100  PF3D7_1358200  PF3D7_1456500 | F  R  F  R  F  R  F  R  F  R  F  R | CATCACCACAGCCAGGATCCGGGAATTAACAAATTGATTGAC  ACTTAAGCATTATGCGGCCGCCCATGGAAACCATGTTC  CACAGCCAGGATCCGAATTCATTTATTTTGTACAAATATAAATAT  ACTTAAGCATTATGCGGCCGCTATTATATTATCTATATTATACTTT  CATCACCACAGCCAGGATCCGGATGTACTTGAAAAAATGC  ACTTAAGCATTATGCGGCCGCCAATAGTGTAGACCTC  CATCACCACAGCCAGGATCCGGGTCCTACAACAATAAATAC  ACTTAAGCATTATGCGGCCGCGCTTTTTCTTGTTCTTTTAC  CATCACCACAGCCAGGATCCGGTGTATTATATATACATGTGG  ACTTAAGCATTATGCGGCCGCCATATGAATAGGAAACCATAC  CACAGCCAGGATCCGAATTCAAGAAAATATACTTTTATATTCTAT  ACTTAAGCATTATGCGGCCGCTATGTGAGATTGGTTAGCTA | pETDuet-1  pETDuet-1  pETDuet-1  pETDuet-1  pETDuet-1  pETDuet-1 | BamHI-NotI  EcoRI-NotI  BamHI-NotI  BamHI-NotI  BamHI-NotI  EcoRI-NotI |
|  |  |  |  |  |  |
|  |  |  |  |  |  |
| *In silico* screening | PF3D7_0304300  PF3D7_0305500 | F  R  F  R | CATCACCACAGCCAGGATCCGGATGGAATAATAAAAGATAGAC  ACTTAAGCATTATGCGGCCGCGAAATTTTCCTTTTGTATGTAAAAG  CATCACCACAGCCAGGATCCGGACTTAAAGTTATATAGAGAG  ACTTAAGCATTATGCGGCCGCCACGGATATATTATATACAC | pETDuet-1  pETDuet-1 | BamHI-NotI  BamHI-NotI |
|  | PF3D7_0305500 | F  R | CATCACCACAGCCAGGATCCGGAAAGAATATACTCACCAAAT  ACTTAAGCATTATGCGGCCGCCATCATATCATTATCATCTG | pETDuet-1 | BamHI-NotI |
|  | PF3D7_0323300  PF3D7_0404700 | F  R  F  R | CATCACCACAGCCAGGATCCGCCTGTATGTTTTAGTACC  ACTTAAGCATTATGCGGCCGCGAAATTCCCTTCATTTAGC  CATCACCACAGCCAGGATCCGCAGAAAGGTGATGTTTCATATAG  ACTTAAGCATTATGCGGCCGCCTGAACAGGAATTTTGTTGG | pETDuet-1  pETDuet-1 | BamHI-NotI  BamHI-NotI |
|  | PF3D7_0407300 | F  R | CATCACCACAGCCAGGATCCGGTAAATAATGATGAAACGTTC  ACTTAAGCATTATGCGGCCGCCATAAATAAATACATATAATCATC | pETDuet-1 | BamHI-NotI |
|  | PF3D7_0416900  PF3D7_0526500 | F  R  F  R | CATCACCACAGCCAGGATCCGGAAATTATAACTACCCAATTAATTC  ACTTAAGCATTATGCGGCCGCGTTATAATTAAGAATATCTTCATTTG  CATCACCACAGCCAGGATCCGGCCATACGTATTATGAGAGAAG  ACTTAAGCATTATGCGGCCGCGTCTCCACATTTATTACCATC | pETDuet-1  pETDuet-1 | BamHI-NotI  BamHI-NotI |
|  | PF3D7_0610100 | F  R | CATCACCACAGCCAGGATCCGCTGAAACATCAAAGGTATAAG  ACTTAAGCATTATGCGGCCGCGTTTTGGTTGTTCATAATATC | pETDuet-1 | BamHI-NotI |
|  | PF3D7_0628100 | F  R | CATCACCACAGCCAGGATCCGGATGCTGTACAAAATGAATAC  ACTTAAGCATTATGCGGCCGCCATTTCTTTTGTATTTGCACTG | pETDuet-1 | BamHI-NotI |
|  | PF3D7_0628100 | F  R | CACAGCCAGGATCCGAATTCGGACATAAAAAAGAAACTACAG  ACTTAAGCATTATGCGGCCGCCATATTTTCATTTTCGTGAGG | pETDuet-1 | EcoRI-NotI |
|  | PF3D7_0723800 | F  R | CATCACCACAGCCAGGATCCGGATAGTATAAATTATAATGATAAG  ACTTAAGCATTATGCGGCCGCCGATTTATCAAGATCTTCC | pETDuet-1 | BamHI-NotI |
|  | PF3D7_0727500 | F  R | CATCACCACAGCCAGGATCCGGGATGTAAGTCAAAAATATATG  ACTTAAGCATTATGCGGCCGCGTTTGTTTTTCTAGATACCTTTAG | pETDuet-1 | BamHI-NotI |
|  | PF3D7_0804500  PF3D7_0913600 | F  R  F  R | CATCACCACAGCCAGGATCCGCTTGTAAATATTCTTTTATATAATTC  ACTTAAGCATTATGCGGCCGCCCTTACATCTTTTAATATCAAC  CATCACCACAGCCAGGATCCGCATAAGCATTTAATATATATTAACG  ACTTAAGCATTATGCGGCCGCCTGTGTACGTAATTTATTTTG | pETDuet-1  pETDuet-1 | BamHI-NotI  BamHI-NotI |
|  | PF3D7_0917500 | F  R | CATCACCACAGCCAGGATCCGGATCATAAAAAGAAAGCACATG  ACTTAAGCATTATGCGGCCGCCTTTTCATTAGATTCATTTGTTG | pETDuet-1 | BamHI-NotI |
|  | PF3D7_0930100 | F  R | CATCACCACAGCCAGGATCCGGAAGCTCAGTCATTTCTTG  ACTTAAGCATTATGCGGCCGCGATATCTATATTTATAGTTTCG | pETDuet-1 | BamHI-NotI |
|  | PF3D7_1003700 | F  R | CATCACCACAGCCAGGATCCGCATATTAAGGATATTTATGC  ACTTAAGCATTATGCGGCCGCCAATTCATATATATCCTTTTC | pETDuet-1 | BamHI-NotI |
|  | PF3D7_1008100 | F  R | CATCACCACAGCCAGGATCCGGATAAATATAAAAGAAGCACAAGAAG  ACTTAAGCATTATGCGGCCGCCATCTTTTCTATGCTTTTTTTAATTTTC | pETDuet-1 | BamHI-NotI |
|  | PF3D7_1029400 | F  R | CATCACCACAGCCAGGATCCGGATAATAAATGTGAAGAAAATG  ACTTAAGCATTATGCGGCCGCCTTCATATCTTTTGGTATATTTA | pETDuet-1 | BamHI-NotI |
|  | PF3D7_1106800  PF3D7_1107700 | F  R  F  R | CACAGCCAGGATCCGAATTCAGAAACAAAAAAATTCAACAACATGG  ACTTAAGCATTATGCGGCCGCACAAGAGGATGAAATGGGAAAGTTGTC  CATCACCACAGCCAGGATCCGCATATAATAAAAGAACGATATCC  ACTTAAGCATTATGCGGCCGCGCTTTGTAGTTCTTGATTCATG | pETDuet-1  pETDuet-1 | EcoRI-NotI  BamHI-NotI |
|  | PF3D7_1109100 | F  R | CATCACCACAGCCAGGATCCGGATATATGTAACAATGTACATG  ACTTAAGCATTATGCGGCCGCGTAACTAATTTTGATAGGTTC | pETDuet-1 | BamHI-NotI |
|  | PF3D7_1113700 | F  R | CATCACCACAGCCAGGATCCGCATTTTAATGAGTATAACTTTTCTC  ACTTAAGCATTATGCGGCCGCCTTATTTGATTGATCTTCTATC | pETDuet-1 | BamHI-NotI |
|  | PF3D7_1200100 | F  R | CATCACCACAGCCAGGATCCGGTGACTGACTATAGTAAAGC  ACTTAAGCATTATGCGGCCGCCTTTTCTAATTTATCATTTTCTC | pETDuet-1 | BamHI-NotI |
|  | PF3D7_1220100 | F  R | CATCACCACAGCCAGGATCCGCCAGATGATACAAATACATTC  ACTTAAGCATTATGCGGCCGCCCATAATTTAATTGTTCCATCC | pETDuet-1 | BamHI-NotI |
|  | PF3D7_1244200 | F  R | CATCACCACAGCCAGGATCCGGATAAAGCAAATTCTCATCTAC  ACTTAAGCATTATGCGGCCGCCGAAAAAGCTTCTCTACTC | pETDuet-1 | BamHI-NotI |
|  | PF3D7_1322100 | F  R | CATCACCACAGCCAGGATCCGCTTAATATAAAAGAATGTACTG  ACTTAAGCATTATGCGGCCGCCTCTTGTAACGATAATAATC | pETDuet-1 | BamHI-NotI |
|  | PF3D7_1364300 | F  R | CATCACCACAGCCAGGATCCGCAAACTATTGGTATGGATGTATTAC  ACTTAAGCATTATGCGGCCGCGCACCAACTATATGAATAATTATG | pETDuet-1 | BamHI-NotI |
|  | PF3D7_1366300 | F  R | CATCACCACAGCCAGGATCCGGGATATGAATCACATCAACATTTAG  ACTTAAGCATTATGCGGCCGCGATATCTGCTTTGTCCTCAAC | pETDuet-1 | BamHI-NotI |
|  | PF3D7_1367500 | F  R | CATCACCACAGCCAGGATCCGGAAGAATTAGACACAAAAGAAAAAAG  ACTTAAGCATTATGCGGCCGCCAAACGAAAGAAAGGAGTCATTC | pETDuet-1 | BamHI-NotI |
|  | PF3D7_1460500 | F  R | CATCACCACAGCCAGGATCCGGGTGGTATATATGATCATAATAATG  ACTTAAGCATTATGCGGCCGCCGGATTCACTGCAACTTTACTC | pETDuet-1 | BamHI-NotI |
|  |  |  |  |  |  |
| RVxF mutagenesis | PF3D7_0220000  PF3D7_0919900 | F  R  F  R | CATCACCACAGCCAGGATCCAGGTTTGGAAGAAGATGATTTAGAAGAAG  CATTATGCGGCCGCAAGCTTATCCTTAATATCAAACCTTACTTACTT  GTATAAAAAAAAGTGCGTCCGCTTTTAAG  CTTAAAAGCGGACGCACTTTTTTTTATAC | pETDuet-1  pQE30 | BamHI-HindIII  BamHI-HindIII |
|  | |  |  |  |  |
